# Evaluation of a multimodal school-based depression and suicide prevention program among Dutch adolescents: design of a cluster-randomized controlled trial

**DOI:** 10.1186/s12888-018-1710-2

**Published:** 2018-05-10

**Authors:** Mandy W. M. Gijzen, Daan H. M. Creemers, Sanne P. A. Rasing, Filip Smit, Rutger C. M. E. Engels

**Affiliations:** 1 Trimbos Institute (Netherlands Institute of Mental Health and Addiction), P.O. Box 725, 3500 AS Utrecht, The Netherlands; 2 Erasmus School of Social and Behavioural Sciences, Erasmus University, P.O. Box 1738, 3000 DR Rotterdam, The Netherlands; 3 GGZ Oost Brabant, P.O. Box 3, 5427 ZG Boekel, The Netherlands; 4 Behavioral Science Institute, Radboud University Nijmegen, P.O. Box 9104, 6500 HE Nijmegen, The Netherlands; 5 Child and Adolescent Studies, Utrecht University, P.O. Box 80140, 3508 TC Utrecht, The Netherlands; 6 Department of Clinical, Neuro and Developmental Psychology and Department of Epidemiology and Biostatistics, Amsterdam Public Health research institute, VU University Medical Center, PO Box 7057, 1007 MB Amsterdam, The Netherlands

**Keywords:** Prevention, Suicide, Depression, Adolescents, School-based, Multimodal

## Abstract

**Background:**

Since 2010, suicide has been the most important cause of mortality in youth aged 15 to 29 years in the Netherlands. Depression is an important risk factor for suicidal behaviors (i.e., suicide ideation, deliberate self-harm, planning, and suicide attempts) in adolescents. Adolescents who develop depressive symptoms, are also at risk for adult depression. This developmental continuity is especially noticeable in adolescents compared to other age groups; therefore, it is necessary to develop preventive strategies for teens. This study will test a multimodal school-based approach to suicide and depression prevention, which integrates universal and targeted approaches and includes various stakeholders (schools, adolescents, parents, and mental health professionals) simultaneously.

**Methods:**

We will perform a cluster randomized controlled trial (RCT) with an intervention and control condition to test the effectiveness of a school-based multimodal stepped-prevention program for depression and suicidal behaviors in adolescents. Adolescents in their second year of secondary education will participate in the study. The participants in the intervention condition will receive the entire multimodal stepped-preventive program comprising early screening and detection of suicidal behaviors and depressive symptoms, a safety net consisting of gatekeepers at school, followed by universal and indicated prevention. The participants in the control condition will undergo only the screening and the safety net of gatekeepers at schools. They will complete assessments at baseline, post-intervention, and 6, 12, and 24-month follow-up. Primary outcome will be suicidal behaviors measured at 12-months follow-up. Additionally, the present study will identify mechanisms that mediate and moderate the program effects and test the effect of the program on various secondary outcomes.

**Discussion:**

If the school-based multimodal stepped-prevention program proves to be effective, it could be implemented in schools on a large scale.

**Trial registration:**

The study is registered in the Dutch Trial Register (NTR6622).

## Background

Each year, 11.2% of Dutch youth have suicidal thoughts and 6.6% attempt suicide or engage in deliberate self-harm [1]. Adolescence is a key period with respect to clinical depression, as the incidence rates of depression rise dramatically from the early to late adolescent years [2]. It is therefore crucial that health care policies focus on preventive interventions that aim to reduce the incidence of depression and suicidal behaviors in early adolescence. We will test a multimodal stepped-prevention program for 12–15 years old and, if effective, implement it in secondary schools in the Netherlands. The program consists of four modules: early screening and detection, gatekeepers training, program targeting stigma, and indicated depression prevention. The current study will examine the overall effectiveness of the multimodal stepped-prevention program for suicidal behaviors and depressive symptoms using a cluster-randomized trial. Through intensive structural collaboration between municipality health services, schools, mental health agencies, and national institutes, we will facilitate an outstanding situation for the implementation of the multimodal stepped-prevention program.

The problem we address is both widespread and severe. In the Dutch population of 17 million, approximately five people per day die from suicide [3]. This affects not only family and friends, but also society. Two studies (in Ireland and Scotland) were conducted to estimate the burden of suicide on society, revealing the cost of €1.5 million per suicide [4, 5]. Suicide before the age of 15 is quite rare, but suicide rates rise substantially during adolescence [2]. To illustrate, suicide is the single most important cause of death among 15 to 29 years old in the Netherlands [3]. It is important to note that suicidal behaviors (i.e., suicidal ideation, deliberate self-harm, and suicide attempts) are often initiated at the age of 15 years; thus, well before a completed suicide [6]. Adolescents themselves recounted that the onset of their suicidal behaviors was when they were 12–16 years old, indicating the need for initiating prevention during early adolescence rather than late adolescence [7]. Therefore, suicidality should in most cases be viewed as a process wherein initial suicidal behaviors remain unnoticed until death by suicide [8, 9]. Suicidal behaviors in young adolescents are sometimes mistaken for behaviors that early adolescents might outgrow [10]. However, approximately one third of adolescents with suicidal ideation will eventually attempt suicide, generally within one year [11]. Moreover, suicidal ideation in adolescence is strongly related to suicidal behaviors in adulthood and is predictive of a range of other adverse outcomes [12–14]. To illustrate, a longitudinal study found that adolescents with suicidal ideation showed significantly more psychopathology and recurrence of suicide attempts and lower perceived coping skills, self-esteem, and social connectedness [15]. Moreover, it appears that suicidal behaviors in adolescents have a high likelihood of recurrence [16]. Adolescents who attempt suicide are more likely to re-attempt suicide compared to adults who attempt suicide [17]. Thus, it is important to recognize adolescent suicidal behaviors at an early stage, take them seriously, and implement strategies to prevent the rise in suicides during adolescence. Studies among adults have indicated that preventive strategies aimed at suicide can indeed reduce suicidal behaviors [18]. Fewer studies have been carried out in adolescents or young adults. Therefore, the aim of this study will be to investigate the effectiveness of a multimodal stepped-prevention program for suicidal behaviors among adolescents, with the aim to implement the program once proven effective.

The existing research has shown that the most common motive for suicide among adolescents is suffering from mental problems [19]. Most adult disorders have their origin in adolescence, with more than three-quarter of disorders starting before the age of 24 [20]. In this context, depression appears to be a significant risk factor for suicidal behaviors in adolescents [21]. This close relationship between depressive symptoms and suicidal behaviors is further established by the fact that the rise of suicidal behaviors in adolescence coincides with increasing incidence of major depression [22]. After all, the first onset of suicidal ideation usually occurs during an episode of depressive disorder in adolescence [21]. In light of this, research suggests that treating depression will likely reduce suicidal behavior as well [23]. Thus, it is important to identify adolescents with subclinical depressive symptoms. Depression is also associated with several negative outcomes. Adolescents with depressive symptoms are more likely to smoke, binge eat [24, 25], and have school-related problems, such as low grades and high drop-out [26, 27]. Once adolescents develop depressive symptoms, they are also at risk for depressive recurrences during adulthood [28]. When a depressive episode initiates during a younger age, the prognosis is far worse than when the first depressive episode initiates during adulthood [29]. This is especially disconcerting considering that depression rates among adolescents have been rising in the past years [30].

In sum, integration of suicide and depression prevention is both a necessary and valuable approach for adolescents. The most relevant setting to reach adolescents is the school, as school attendance is mandatory in the Netherlands until the age of 18. Few school-based prevention programs actually address suicide prevention. Yet, there are some school-based programs aimed at depression prevention have been proven effective in Dutch samples [31, 32]. Current approaches to depression and suicide prevention at schools comprise mostly singular interventions. Growing evidence suggests that depression and suicide prevention warrant the adoption of a multimodal approach to become truly effective [33]. The lack of an integrated multimodal approach is also evident from the available intervention options in the National Institute for Public Health and the Environment (in Dutch: Rijksinstituut voor Volksgezondheid en Milieu, RIVM) and Centre for Healthy Living (in Dutch: Centrum Gezond Leven, CGL) database as well as the healthy school database in the Netherlands [34–36]. Although several programs focus on the recognition of risk factors for suicide or for the prevention of depression, none of these options offer a comprehensive multimodal approach targeting both depression and suicide prevention. Integrating different complementary types of prevention (i.e., universal and indicated approaches) that would include various stakeholders simultaneously (e.g., teachers, adolescents, parents, youth (mental) health service providers) and using various types of interventions (screening, education, universal, and indicated interventions) have been suggested as prevention strategies.

The current study will examine the effectiveness of the multimodal stepped-prevention program for suicidal behaviors and depressive symptoms using a cluster randomized trial. This includes a combination of preventive interventions, such as (1) early screening and detection of suicidal behaviors with subsequent clinical referral, (2) a safety net consisting of gatekeepers at school, (3) universal prevention focusing on stigma reduction, and (4) identification of adolescents who have elevated signs of depression with subsequent indicated prevention. Early detection is important, as less than half of adolescents engaging in suicidal behaviors are known at mental health care services or by other gatekeepers (i.e., family, friends, teachers and mentors at school, etc.) prior to a suicide [37, 38]. A growing body of evidence suggests that school-based screening adequately identifies students at high-risk, effectively refers these students to mental health care, and reduces the risk of suicide ideation and non-fatal suicidal behaviors [39, 40]. It is similarly important that a safety net is created at schools. Mentors should have the knowledge and skills to identify adolescents who show signs of suicidal behaviors and know how to respond to those students [41]. Previous research has shown that a gatekeeper training based on Question, Persuade, and Refer (QPR) model can increase knowledge of suicide prevention and skills [42, 43]. Another important factor impeding identification of suicidal behaviors is the fact that help-seeking behaviors among youths is very low [44]. Nevertheless, research has shown that help-seeking behaviors predicts better prognosis [45]. Stigma has been identified as an important factor that impedes help-seeking among youth. Thus, it is important to develop a universal strategy aimed at reducing stigma.

Stigma literature suggests that mental health literacy combined with information related to individuals’ personal experience is more likely to produce a change in attitude and stigma [46]. Moreover, contact with someone who has personal experience with depression seems to be a crucial factor for changing stigmatizing attitudes [47]. This true not only for students not showing signs of depression or suicidal behaviors, but also for those with mental health problems (e.g., suicidal behaviors or depressive symptoms) when they identify with others suffering from mental health problems [48].

The identification of and subsequent intervention for those with elevated signs of depressive symptomatology should also be important components of the prevention of suicidal behaviors [21]. Cognitive behavioral therapy (CBT) is an effective treatment for depression, and many indicated prevention programs are based on CBT, such as ‘Op Volle Kracht (On Full Power: OVK). OVK was modeled after the Penn Resiliency Program (PRP) [49]. The program successfully reduced depressive symptoms in adolescent girls with subclinical depressive symptoms [50]. A modified OVK version (OVK2.0) by de Jonge-Heesen, et al. [51] will be implemented and investigated at schools with adolescents who have been identified as scoring higher on depressive symptoms, as measured by self-report.

The primary aim of our study will be to investigate the effectiveness of a multimodal stepped-prevention program to reduce suicidal behaviors and depressive symptoms. We hypothesize that suicidal behaviors will decline as a result of a multimodal preventive intervention relative to care as usual enhanced with screening and the gatekeepers’ safety net. Since most modules of the stepped-prevention program are based on depression prevention program, we expect that depressive symptoms will decline as well. Secondary aims are to investigate the mechanisms of change by studying the effect mediators and moderators. Previous research has identified several factors that contribute to lower treatment response in depression and suicidal behaviors. Socio-demographic factors, such as age, gender, cultural background/ethnicity, and educational background, have been found to moderate treatment outcome. Therefore, their effects will be examined. Other factors that could influence treatment outcome are perfectionism, hopelessness, and baseline depressive symptoms and suicidal behaviors. Furthermore, other factors that are related to the proposed prevention modules could mediate outcome, such as stigma, social connectedness, mastery, worry, and life events. We hypothesize that the factors that impede treatment response will have a similar effect on the outcomes of the multimodal stepped prevention program.

## Methods

The study methods and results will be reported in accordance with the CONSORT 2010 statement for reporting parallel group randomized trials [52] and the CONSORT 2010 statement: extension to cluster randomized trials [53]. The medical research ethics committee CMO Region Arnhem-Nijmegen in The Netherlands approved this study (NL61599.091.17). The study is registered in the Dutch Trial Register (NTR6622).

### Design

**Fig. 1:**
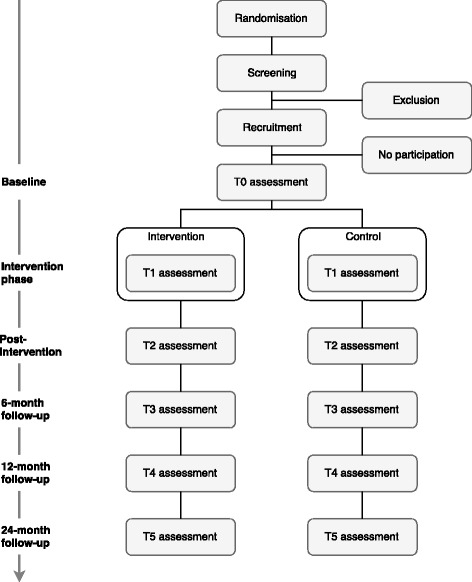
Schematic Overview of the Study Design

The presented study is designed as a non-blinded cluster-randomized controlled trial with two parallel groups (experimental and control) to evaluate the effectiveness of a multimodal stepped-prevention program relative to (enhanced) usual care. The participants in the experimental condition will receive all four modules of the multimodal stepped-prevention program, whereas participants in the control condition will receive modules 1 and 2. Randomization will be conducted at school level (to be more precise: at the level of school location because one school can have annexes at multiple locations) to avoid contamination. An independent statistician will randomly assign the participating school locations to the intervention or control condition using random.org. Furthermore, randomization will be stratified for (1) education level (vocational training as one category, in Dutch: VMBO; and higher education / pre-university as the other category, in Dutch: HAVO/VWO) because school type is a relevant prognostic predictor of outcome [54]. Like most psychological and public health interventions, blinding of participants is not possible; therefore, it will not be attempted. The assessments will be conducted at baseline (T0), during the intervention phase after the third module (T1), at post-intervention (T2), at 6-month follow-up (T3), at 12-month follow-up (T4), and at 24-month follow-up (T5). The overall study design is shown in Fig. 1.

### Participants’ eligibility

Adolescents in their second year of secondary education will be eligible to participate in the study. Inclusion criteria are aged between 11 and 15 years and sufficient knowledge of the Dutch language. Exclusion criteria are absence of (parental) permission and for the indicated module (OVK2.0) already receiving health care for mood-related problems.

### Recruitment

Following the randomization at the school level, as mentioned above, students in their second year of secondary school (i.e., 8th grade in the US), from vocational training up to pre-university level, will receive written information about the screening and the study. Written informed consent from adolescents and their parents will be obtained prior to the initiation of the study.

### Sample size

We powered our study to detect a standardized mean difference *d* = 0.38 (or larger) on the central clinical outcome (suicidal behaviors) using a test for independent means at α = 0.05 (2-tailed) and power of (1-b) = 0.80 while considering the design effect of the cluster randomization with schools as the unit of randomization and students being nested within schools. The relevant parameters are (1) the intraclass correlation coefficient, rho; the mean cluster size, *m*; the coefficient of variation, *cv*, of the cluster sizes; and the minimally detectable effect size, *d*. Regarding the last parameter, we looked at the well-known SEYLE study [55], where 2764 pupils in 43 schools were randomized to a universal prevention consisting of a Youth Aware of Mental Health (YAM) program (comparable to our universal intervention) and compared to a control group of 2933 students in 40 schools. YAM was associated with a significant reduction in incident suicide attempts (OR = 0.45, *p* = 0.014) and a reduction in severe suicidal ideation (OR = 0.50, *p* = 0.025). In our study, we expect similar or better effects because in addition to the universal preventive module, we will also offer the safety net provided by the gatekeepers training in addition to early detection and referral for adolescent students at elevated risk of suicide. For screen-positive students, an additional indicated preventive module will be offered. Nonetheless, we conservatively assume that the effectiveness of our integrated multi-component intervention will be of equal effectiveness as the YAM program. The OR of 0.50 translates into a standardized mean difference, *d*, of 0.38 when the outcome is measured on a continuous scale, as we will do [56]. The mean cluster size of *m* = 215 will be based on student population in the previous year. In the last year, the smallest school had 74 students and the largest 340. Accordingly, it follows that coefficient of variation *cv* = 0.31 [57]. In the SEYLE study of Wasserman et al. [55], the intraclass correlation coefficient was estimated at rho = 0.01 for severe suicidal ideation. To obtain an estimate of the required sample size, we used Stata’s procedure to estimate sample size needed for an independent groups t-test in a cluster randomized trial [58]. The corresponding syntax of clustersampsi, samplesize mu1(0.00) mu2(0.38) sd1(1) sd2(1) rho(0.01) m(215) size_cv(0.31) indicated *n* = 645 per condition, or *n* = 1290 in both trial arms. We will also compensate for a maximum of 30% dropout, which implies that we will need to have 1290/(1–0.30) =1844 participants at baseline.

### Program modules

The multimodal stepped-prevention program comprises screening with subsequent clinical evaluation and/or referral; gatekeeper training (QPR) for mentors; universal prevention focusing on stigma reduction; and identifying adolescents who have elevated signs of the most important risk factor for suicidal behaviors, i.e., depression with subsequent indicated prevention for screen-positive adolescents. These integrated four modules will be compared to the control condition. Schools in the control condition will adhere to the usual curricula and students in these schools will have full access to usual care offered by the regional health services, e.g., the Municipal Health Services (GGD), Primary Care, and (specialized) Mental Health Care services. In the context of this trial, we will prioritize the wellbeing and safety of participating students, especially those at elevated risk for suicidal behaviors and/or depressive disorder. Therefore, the screening and gatekeeper intervention will also be available to the control group. Consequently, the comparison condition will not exactly be “care as usual”; instead, it is described as “enhanced usual care” to guarantee the wellbeing and safety of participating students.

#### I. Screening

All students in their second year of secondary school (i.e., 8th grade in US), from vocational up to pre-university levels, will be screened for suicidal behaviors and depressive symptoms using the Questionnaire assessing Suicide and Self Injury (in Dutch: Vragenlijst over Zelfdoding en Zelfbeschadiging; VOZZ) [59] and the Childhood Depression Inventory 2 (CDI-2) [60]. The screening will be part of a larger health survey conducted by the health services of the school (in Dutch: GGD). Adolescents identified at risk for suicide are seen within 48 h by the health service of school (in Dutch: GGD). Parents of children identified as at risk for suicide by the mental health service of school will be informed. In accordance with parents, the adolescents will be referred to specialized mental health care, if deemed necessary by the health service of school, and will be excluded from the indicated intervention module. Adolescents who have been referred to specialized mental health care will also be approached to complete the same set of questionnaires as the children in the experimental and control condition to examine the effect of the screening and to monitor their mental health.

#### II. Gatekeeper training

All mentors of participating adolescents will be used as gatekeepers and trained to recognize suicidal behaviors, learn to initiate conversations after recognizing suicidal behaviors in a student, and learn to refer a student effectively and correctly, if deemed necessary. This training will be based on the Question, Persuade, and Refer (QPR) gatekeeper training [61]. Gatekeepers who will complete the QPR will show increased knowledge of suicide prevention, self-reported skills, competencies, and efficacy [42, 43]. Further information on the content, background, and structure of the online program is provided through an open-access protocol paper [62].

#### III. Universal prevention

The third module that will be evaluated is ‘Moving Stories’, which will be offered to all participating students in the experimental condition. The goal of ‘Moving Stories’ is to increase students’ mental health literacy (i.e., knowledge of depressive symptoms, effective strategies for dealing with depressive symptoms, and help-seeking strategies) and decrease depression stigma. It consists of an introduction presented by a researcher and the students’ school mentor, a serious game, and a debriefing by either an experiential expert only or a mental health professional in combination with a filmed experiential expert trained in the program with support from the students’ school mentor and a researcher at school. The game consists of five sessions, approximately 10–15 min per day. In a virtual house, adolescents will be asked to discover useful strategies to help a girl, Lisa, who is showing signs of depression. They can complete five actions each day and earn points with these actions. More points represent more trust between Lisa and the player, and this enables the players to call in help from an adult. For example, students can make drinks or food, call her parents, or clean the house, among other things. To identify useful strategies, they can talk to the girl in the house. Students will receive feedback on their actions during the day.

#### IV. Indicated prevention

The fourth module, namely the indicated module, is ‘OVK2.0’, which will only be offered to screen-positive students (CDI-2 ≥ 14) in the experimental condition. It consists of 8 lessons of 60 min each. The intervention is based on the principles of cognitive behavioral therapy (CBT) and is a modified version of the OVK-program that was based on the PRP. It only includes the lessons that are based on the CBT techniques, as they were considered most effective [50]. Details about the content of the program are described extensively elsewhere [49]. It will be delivered by two trainers, a licensed psychologist who is a school staff member and a co-trainer who is a licensed member of a (mental) health institution. The trainers underwent an extensive 3-day training program in the necessary skills, such as CBT and its theoretical background, a training manual, and the intervention protocol.

### Study outcome measures

For an elaborate overview of study outcome measures, see Table 1.

**Table 1:** Overview of assessments

	Screening	T0	T1	T2	T3	T4	T5
Adolescent							
Suicide risk (VOZZ-Screen)	X						
Suicidal behaviors (VOZZ)		X	X	X	X	X	X
Depressive symptoms (CDI-2)	X	X	X	X	X	X	X
Clinical depression (ADIS-C)						X	
Stigma (DSS)		X	X	X	X	X	X
Social connectedness		X	X	X	X	X	X
Mastery (PMS)		X	X	X	X	X	X
Worry (PSWQ)		X	X	X	X	X	X
Perfectionism (MPS)		X					
Life events (TP)		X	X	X	X	X	X
School							
Academic grades	X	X	X	X	X	X	X
Drop-out rates	X	X	X	X	X	X	X
Non-attendance	X	X	X	X	X	X	X
Truancy	X	X	X	X	X	X	X

### Screening

To assess suicide risk, adolescents will be screened using the VOZZ [59] and item 8 of the CDI-2 [60]. The VOZZ-Screen is a self-report questionnaire comprising 10 items. This questionnaire contains ten questions assessing thoughts and actions about life, self-harm, suicide, and suicidal ideations in the past 7 days. Items assessing the participant’s life are rated on a 5-point scale from 1 (I totally agree) to 5 (I totally disagree) (e.g., ‘I feel worthless’). Items about self-harm and suicide are rated on a 5-point scale from 1 (never) to 5 (very often) (e.g., ‘I have harmed myself deliberately’). Items about suicidal ideation in the past 7 days are rated on a 5-point scale from 1 (never) to 5 (every day) (e.g., ‘I thought that suicide would be a solution for my problems’). A score of ≥23 requires subsequent action in the form of a personal conversation to assess acute suicide risk. The CDI-2 is also a self-report questionnaire comprising 28 items assessing depressive symptoms, each consisting of three statements rated in severity from 0 to 2 (e.g., ‘I don’t feel alone’ = 0 ‘I often feel alone’ = 1, ‘I always feel alone’ = 2). Item 8 of the CDI-2 measures the presence of suicidal ideation on a three-point scale (0 = I don’t think about ending my life, 1 = I think about ending my life, but I would never do it, 2 = I want to end my life). The CDI-2 will be used for screening purposes in accordance with the Dutch clinical guidelines for depression among youth [63].

### Primary outcome measure

*Suicidal behaviors* will be measured using the full VOZZ questionnaire [59]. This questionnaire contains 39 questions assessing thoughts and actions about life, self-harm, suicide, and suicidal ideations in the past 7 days. It is a combination of the VOZZ-Screen and 29 additional items. Scoring is the same as described earlier for the VOZZ-Screen. A score of 86 or above indicates high risk of suicide. The reliability was high in an adolescent sample (Cronbach’s α = 0.91; *r* = 0.82) [59].

### Secondary outcome measures

*Depressive symptoms* in children and adolescents will also be measured with the CDI-2 [60], as described in previous section.

*Clinical depression* will be measured by the Anxiety Disorder Interview Schedule for Children (ADIS-C; [54]) during a clinical interview. It is a semi-structured diagnostic interview that is used to diagnose anxiety and comorbidity. All interviews will be administered by a qualified psychologist.

*Stigma* will be measured using the Depression Stigma Scale (DSS) [64]. It measures personal depression stigma and perceived depression stigma. Both scales have a good internal consistency (Perceived Scale: Cronbach’s α = 0.82; Personal Scale: Cronbach’s α = 0.78) [64]. The Personal Stigma Subscale measures stigma in the respondents’ own attitudes towards depression. The Perceived Stigma Subscale measures the respondent’s perception about the attitudes of others towards depression. Both subscales consist of 6 items and responses to each item are measured on a five-point scale (ranging from 0 ‘strongly disagree’ to 4 ‘strongly agree’). Higher scores indicate higher levels of depression stigma.

*Social connectedness* will be measured using a single item construct. Research has shown that a single item measure of social identification is reliable [65]. As the construct has not yet been used in this age group, we have included three extra items for (potential) increased reliability of the construct. Social connectedness will be measured for the class, the ‘OVK’-group, and school. Items are rated on a scale of 1 (“strongly disagree”) to 7 (“strongly agree”).

*Mastery* will be measured with the Pearlin Mastery Scale (PMS; [66]), which consists of 7 items. It measures perceived control of one’s life. Each item has the following response options: (1) Strongly Disagree (2) Disagree (3) Agree (4) Strongly Agree. It is a widely used measurement of mastery, with a higher score indicating higher mastery. Previous studies have found good reliability of the PMS (Cronbach’s α = 0.78) [67].

*Worry* will be assessed with the Penn State Worry Questionnaire for children (PSWQ-C; [68]). It is widely used in both research and clinical practice to reliably assess worry in both clinical and non-clinical samples and adolescents [69]. The Dutch version has also been shown to be reliable for assessing worry in children [70]. The PSWQ-C consists of 14 items. Each item is rated on a scale of 0 (“never”) to 3 (“always”). Higher scores indicate higher levels of worry.

*Perfectionism* will be assessed using the Frost Multidimensional Perfectionism Scale (FMPS) [71]. It consists of 35 items and has good internal reliability (Cronbach’s α = 0.90) [71]. Each item is rated on a scale of 1 (not true at all) to 5 (completely true), with a higher score indicating more levels of perfectionism. It measures six subscales, namely concern over mistakes, personal standards, parent expectations, parental criticism, doubting of actions and organization. A higher score indicates greater levels of perfectionism.

*Life events* will be recorded and assessed using The Top Problems (TP) [72] measure. It assesses life events that participants consider the most important at that time. Participants are asked to list three problems about which they are most concerned. Furthermore, they are also asked to rate the severity of all three problems separately on a scale from 0 (not at all) to 10 (very, very much). As such, it not only measures actual life events, but also accounts for personal salience of life events, as opposed to most life events measures.

*Hopelessness* will be assessed using the VOZZ. Previous studies have found that correlations between the hopelessness scale by Beck and the VOZZ were 0.79 and 0.58 [59]. The items 3, 4, 21, 28, and 31 of the VOZZ, which were established in accordance with the first author of the VOZZ, will be used to assess hopelessness.

*School-related factors,* such as academic grades, drop-outs, non-attendance, and truancy will be obtained in collaboration with the schools.

### Analysis

The targeted clinical outcome (VOZZ suicidal behaviors) will be evaluated in agreement with the intention-to-treat principle using linear mixed modeling with VOZZ at baseline as covariate. Reporting of the results will be conducted in accordance with the CONSORT statement [52].

#### Evaluation of secondary outcome

The intervention’s effect on the secondary outcome (CDI-2 depression) will be investigated in the same way as the primary outcome (VOZZ suicidal behaviors); hence, using linear mixed modeling with baseline CDI-2 depression as a covariate.

#### Analysis of effect mediation

It is important to determine the mediators that affect the intervention effect. This helps determine whether the intervention works through expected mechanisms and which improvements could be effective. Mediation analyses will be performed in Mplus [73], where indirect effects will be tested with bootstrap methods. More specifically, we will test whether (1) mastery, (2) stigma, (3) worry, (4) top problems, (5) social connectedness, and (6) hopelessness will mediate the intervention effect on VOZZ suicidal behaviors and / or CDI-2 depression.

Increased awareness through stigma reduction will be the main intervention target of the universal prevention. We hypothesize that reduction in stigma will also reduce suicidal behaviors and depressive symptomatology and mediate the effect of the intervention. Moreover, stigma is also related to social isolation, which is another important risk factor for suicidal behaviors [74]. Research has found that people often withdraw from social life due to fear of rejection as a result of stigmatizing attitudes towards mental health problems [75, 76]. Hence, reduced stigma is likely to reduce social isolation. Previous research has also shown that students who have negative attitudes towards school and thus feel less connected to the school are at an increased risk for suicide attempts [77]. School-based programs are expected to induce attitude changes in school staff and students. Moreover, group-based interventions could reduce the feeling among adolescents that they are the only ones experiencing depression, which is often associated with adolescent depression, as youngsters often do not share depressive feelings with each other due to fear of stigmatization. Furthermore, social isolation can also reduce treatment response [78]. In line with this, studies have shown that each social group that a depressed individual joins decreases relapse rates in depressed individuals and results in a more pronounced improvement on depressive complaints [79]. Thus, social connectedness is an interesting factor to examine as a potential moderator considering both depression and suicide prevention. Both the interpersonal theory [80] and the integrated-motivational-volitional model [81] theorize that reduced social connectedness increases suicidal ideation through the sense of thwarted belongingness. Christensen [82] found that mastery is also related to the sense of thwarted belongingness and in accordance with previous research, the researcher has found that lower levels of mastery are significantly associated with suicide ideation [83]. In theory, mastery ensures that people have the ability to manage negative experiences [84]. Those who feel lack of control (i.e., mastery) over situations are more likely to turn to suicidal ideation, much like a self-fulfilling prophecy. Moreover, suicidal ideation may give people a false sense of mastery when they feel they lack mastery naturally [85].

In addition to social connectedness and mastery, repetitive thinking is also suggested as a mechanism affecting depression and suicidal ideation [86]. Research has found that repetitive thinking predicts not only presence of depression and suicidal ideation, but also their duration [87, 88]. Furthermore, repetitive thinking decelerates the recovery after the treatment [89–91]. In line with this, Kerkhof and van Spijker [92] identified worrying (i.e., repetitive thinking) as a proximal risk factor of suicidal behaviors. Interestingly, mastery has been associated with negative effect of repetitive thinking [93], which may also be an important mediator, as mentioned before. Another interesting finding is that hopelessness may partially mediate the relationship between repetitive thinking and suicidal ideation [94]. Hopelessness has also often been named as one of the most important predictors of suicide attempts and behaviors [95–98]. Moreover, it is considered to mediate the relationship between depression and suicidal behaviors [95]. Perfectionism is often related to the feelings of hopelessness [99]. Just like those with lower levels of mastery, a person with high feelings of perfectionism may be more likely to turn to suicidal ideation in line of negative events or feelings of hopelessness [100, 101]. As opposed to mastery, perfectionism is traditionally viewed as a more stable trait [102]. Thus, we hypothesize that perfectionism may moderate rather than mediate the treatment outcome. In addition, it is also important to record life events, as they can increase the risk of depressive symptomatology [103]. Negative life events are also associated with the occurrence of suicidal ideation [97]. However, the most important variable is the experienced disruptiveness of life events. As such, recurrences in depression are often associated with negative life events due to kindling and sensitization [104–106]. We expect that the multimodal stepped-prevention program will also reduce the disruptiveness of life events and in turn decrease occurrence of depressive symptoms.

#### Explorative analysis of effect moderation

Finally, moderation analyses will be conducted to determine which adolescents benefit most from the intervention. Additionally, it will help determine whether some adolescents would be better served by receiving adapted or other interventions. We will conduct a series of a priori planned moderator analyses to see whether the intervention effect is moderated (increased or diminished) by the following moderators: (1) gender, (2) ethnic descent / cultural background, (3) level of baseline perfectionism, (4) level of baseline VOZZ suicidal behaviors, and (5) level of baseline CDI-2 depression. We call these analyses explorative because the data-analytical strategies will be non-parametric in nature, such as bootstrap-aggregated CART analysis and random forest methods implemented in the R statistical package.

Several socio-demographic factors have been found to affect suicide and depression prevention. Previous research has found that gender may play an important role in CBT-based programs. Age has also been found to influence treatment outcome in depression prevention studies. Girls and older participants were found to experience more beneficial effects of interventions. It is also important to consider ethnic and cultural background. It has been well established that ethnicity influences help-seeking behaviors and the ways in which suicidal behaviors or depressive symptoms are expressed [107]. Hence, ethnicity might in turn also influence the perception and integration of treatment.

#### Other study parameters

Possible baseline imbalances between the two conditions in demographic variables, VOZZ suicidal behaviors, and CDI-2 depression will be verified. If any variables show different distributions across the two conditions, they will be entered as covariates in all models testing the effectiveness of the intervention.

### Interim analysis

A planned interim analysis will be conducted to assess whether one of the trial’s conditions (either intervention or control) is associated with a significantly higher risk of completed suicides. An independent statistician, blinded to treatment allocation, will carry out the interim analysis at the post measurement. We chose the post measurement as the time point for the interim analysis because it is the earliest stage in the trial to test whether one of the conditions is associated with a significantly greater number of completed suicides. The interim analysis may result in changes in the study’s protocol or the study might even end due to overwhelming evidence of group difference.

The interim analyses will be conducted for completed suicides at the primary efficacy end point of the study obtained from patients in the target population. The appropriate analysis of such count-data (non-negative integers) is best done with Poisson regression, which is also appropriate for analyzing rare events. The statistical analyses will be carried out by setting the alpha-level to 0.05 for a two-tailed test. The Type I error boundaries for statistical significance do not need to be adjusted for multiple comparisons because the interim analysis will be conducted at a single time point.

## Discussion

The present study protocol describes a RCT on the effect of a multimodal school-based prevention program on suicidal behaviors in adolescents. The primary aim is to investigate whether the multimodal school-based program results in a clinically significant reduction of suicidal behaviors and depressive symptom levels in secondary school students compared to ‘enhanced’ care as usual. The secondary aim is the evaluate pathways that transmit the intervention effect on the primary outcome: (a change in) stigma, social connectedness, mastery (i.e., level of internal locus of control), worry, hopelessness, and number of self-reported major problems. Another secondary aim is to identify modifying factors that increase or decrease the intervention’s effectiveness: baseline level op depressive symptoms, baseline level of suicidal behaviors, perfectionism, gender, mastery, and ethnic descent / cultural background.

### Strengths and limitations

One of the strengths of this study is that it will include the follow-up assessment of 24 months, providing the opportunity to evaluate the longer-term effects. Second, the multimodal stepped-prevention program will be implemented in all secondary schools in a rural region in The Netherlands, with a strong collaboration between schools’ and (mental) health organizations. A meta-analysis of Brunwasser and Garber [108] on the effectiveness of programs for the prevention of youth depression revealed the need to conduct studies in real-life conditions, like the one we are proposing. Third, the current study uses a between-schools design which minimizes contamination effects that might occur in a within-schools design. An additional strength of the study is that in contrast to the most RCT studies, we will focus not only on the effectiveness of the program, but also on the mediators of change (i.e., how the intervention works) and on the characteristics of the student population that may act moderators. This will shed light on how the intervention works and for whom it is effective.

Several limitations of this study must be noted. As we evaluate the effectiveness of the multimodal stepped-prevention program, no conclusions regarding the specific components of the program can be made. Additionally, we will not complete a clinical interview prior to the interventions (only at post measurement). Thus, it is possible that some adolescents meeting the diagnostic criteria of a full-fledged depression may be included in the study. For those, the intervention becomes the treatment rather than the prevention. However, since sub-threshold depression or minor depression is sometimes viewed as a clinical disorder (as is the case in the DSM-5), the current study might not be classified as the prevention of the imminent onset of a new disorder, but rather as the treatment of an existing disorder. Additionally, the study will be conducted in a specific region in The Netherlands, which may limit the generalizability of the results to other regions in The Netherlands.

### Implications for practice

If the multimodal stepped-prevention program proves to be effective in reducing suicidal behaviors and preventing depressive symptoms in adolescents, then this will call for a broader implementation of school-based suicide and depression prevention. Moreover, considering all the stakeholders involved in this preventive program, their strong collaboration could benefit the region and serve as an example for other regions in Europe of how to organize suicide and depression prevention for youth.

## Abbreviations

ADIS-C: Anxiety disorders interview schedule for children
CBT: Cognitive behavioral therapy
CDI-2: Children’s depression inventory 2
CMO: Commissie Mensgebonden Onderzoek (in English: Committee on Research Involving Human Subjects)
DSS: Depression Stigma Scale
FMPS: Frost Multidimensional Perfectionism Scale
OVK: Op Volle Kracht (in English: On Full Power)
PI: Principal investigator
PMS: Pearlin Mastery Scale
PRP: Penn Resiliency Program
PSWQ-C: Penn State Worry Questionnaire for Children
QPR: Question, Persuade and Refer
RCT: Randomized controlled trial
TP: Top Problems
VOZZ: Vragenlijst over Zelfdoding en Zelfbeschadiging (in English: Questionnaire assessing Suicide and Self Injury)
YAM: Youth Aware of Mental Health
